# Biology of the SARS-CoV-2 Coronavirus

**DOI:** 10.1134/S0006297922120215

**Published:** 2023-01-13

**Authors:** Rimma N. Mingaleeva, Nigina A. Nigmatulina, Liliya M. Sharafetdinova, Albina M. Romozanova, Aida G. Gabdoulkhakova, Yuliya V. Filina, Rafael F. Shavaliyev, Albert A. Rizvanov, Regina R. Miftakhova

**Affiliations:** 1 Federal State Autonomous Educational Institution of Higher Education “Kazan (Volga Region) Federal University”, 420008 Kazan, Russia; 2 State Autonomous Public Health Institution “Republican Clinical Hospital”, Ministry of Health of the Republic of Tatarstan, 420064 Kazan, Russia

**Keywords:** SARS-CoV-2, COVID-19, S protein, mutation, VOC

## Abstract

New coronavirus infection causing COVID-19, which was first reported in late 2019 in China, initiated severe social and economic crisis that affected the whole world. High frequency of the errors in replication of RNA viruses, zoonotic nature of transmission, and high transmissibility allowed betacoronaviruses to cause the third pandemic in the world since the beginning of 2003: SARS-CoV in 2003, MERS-CoV in 2012, and SARS-CoV-2 in 2019. The latest pandemic united scientific community and served as a powerful impetus in the study of biology of coronaviruses: new routes of virus penetration into the human cells were identified, features of the replication cycle were studied, and new functions of coronavirus proteins were elucidated. It should be recognized that the pandemic was accompanied by the need to obtain and publish results within a short time, which led to the emergence of an array of conflicting data and low reproducibility of research results. We systematized and analyzed scientific literature, filtered the results according to reliability of the methods of analysis used, and prepared a review describing molecular mechanisms of functioning of the SARS-CoV-2 coronavirus. This review considers organization of the genome of the SARS-CoV-2 virus, mechanisms of its gene expression and entry of the virus into the cell, provides information on key mutations that characterize different variants of the virus, and their contribution to pathogenesis of the disease.

## INTRODUCTION

Coronaviruses are defined as a group of related RNA viruses that infect mammals and birds. When infecting humans, these viruses cause respiratory diseases of varying severity, from upper respiratory tract infections similar to those of seasonal colds to severe lower respiratory tract infections, including bronchitis, pneumonia, and SARS (severe acute respiratory syndrome). Avian infectious bronchitis virus (IBV) was the first coronavirus to be discovered [1]. The human-infecting coronaviruses, HCoV-229E and HCoV-OC43, were first identified in 1966 and 1967. In 2003, the zoonotic coronavirus SARS-CoV was discovered in China, its spread led to an epidemic with 8000 documented cases with 10%-fatality rate. This led to a surge of keen interest in coronaviruses, which subsequently resulted in identification of two more viruses: HCoV-NL63 (Netherlands, 2004) and HCoV-HKU1 (Hong Kong, 2004), which circulate annually around the world. In 2012, a second highly pathogenic zoonotic coronavirus, MERS (Middle East respiratory syndrome)-CoV, was identified in Saudi Arabia and is still occasionally detected in humans. MERS-CoV has been confirmed in 2591 cases until July 2022. It affects lungs and causes severe clinical manifestations. Lethality rate of the disease is up to 35% (World Health Organization, https://www.who.int/).

SARS-CoV-2, which causes COronaVIrus Disease 2019 (COVID-19), was the seventh coronavirus discovered to affect humans. According to the Johns Hopkins University Coronavirus Resource Center (https://coronavirus.jhu.edu/map.html), number of the cases of COVID-19 as of September 2022 was 612 million people, of whom 6.5 million died. In Russia, as of March 2022, COVID-19 has been registered in 20 million people, 378 thousand people died (fatality rate is 1.9%) (Johns Hopkins University Coronavirus Resource Center, https://coronavirus.jhu.edu/map.html). Scale of the spread of SARS-CoV-2, ease of transmission of the virus from person to person, alleged existence of intermediate interspecific forms of new coronaviruses dictates the need to develop new methods for diagnosis and treatment of the disease [2].

## CORONAVIRUS FAMILY

Coronaviruses belong to the order Nidovirales, family Coronaviridae, and subfamily Orthocoronavirinae. Orthocoronavirinae consists of four genera: *Alphacoronaviruses*, *Betacoronaviruses*, *Gammacoronaviruses*, and *Deltacoronaviruses*. *Alphacoronaviruses* and *Betacoronaviruses* exclusively infect mammals, while *Gammacoronaviruses* and *Deltacoronaviruses* have a wider range of hosts, including birds [3]. HCoV-229E and HCoV-NL63 are *Alphacoronaviruses*; HCoV-HKU1, SARS-CoV, MERS-CoV, HCoV-OC43, and SARS-CoV-2 are *Betacoronaviruses*.

Many cases of interspecies transmission of coronavirus infection between mammals have been described for the veterinary important viruses. Coronavirus that infects dogs (CCoV), cats (FCoV), and coronavirus that causes transmissible gastroenteritis in pigs (TGEV) are believed to share the same prototype, *Alphacoronavirus 1*. It has been shown that the outbreak of porcine acute diarrhea syndrome (SADS) was caused by the SADS-CoV coronavirus, descendant of the BatCoV-HKU2 alpha-coronavirus found in bats. Similarity between the alpaca coronavirus (ACoV), which is also an *Alphacoronavirus*, and the human virus HCoV-229E, suggests zoonotic origin for the latter. HCoV-OC43 is thought to have originated from a bovine coronavirus (BCoV) around 1890s [4].

Cats, ferrets, dogs, and other mammals not only become infected with coronaviruses specific to their species, but also become infected with SARS-CoV-2 [4]. It is believed that SARS-CoV and SARS-CoV-2 entered human population from bats through intermediate hosts. High degree of homology of the genome of the palm civet coronaviruses and SARS-CoV indicates high probability of the virus transmission to humans [5]. The origin of SARS-CoV-2 is more controversial. Intermediate hosts for this virus could have been pangolins or other animal species, but it is possible that the virus passed to humans directly from bats [6]. The entire receptor-binding motif of the SARS-CoV-2 virus could be introduced by recombination with the pangolin coronaviruses [7]. Recent studies have shown that the SARS-CoV-2 genome may be of mosaic nature that combines genomes of three bat coronaviruses (RmYN02, RpYN06, and RaTG13) found in the Hubei Province of China (capital Wuhan) [8]. In general, there are over 1300 species of bats that form huge flocks (up to several million individuals in a group), travel long distances, and are present on all continents acquiring and spreading many viruses. Among the identified bat viruses, at least 60 may be pathogenic to humans [9]. It is believed that the history of the origin of most human coronaviruses can be traced back to bat viruses. This assumption is based on two facts: (i) diversity and abundance of bat coronaviruses far exceed diversity of these viruses in other mammals; (ii) bat coronaviruses that are very similar to human viruses identified so far: Bat-229E-like, Bat-NL63-like, and Bat-SARS-like viruses. The probability of these viruses jumping from species to species is determined by (i) compatibility of the virus with the receptors located on the cells of the host organism (cell susceptibility); (ii) ability of the virus to replicate in the cells of the host organism (cell permissiveness); (iii) availability of the cells susceptible to the virus; (iv) ability of the virus to evade the host’s immune response. The Spike viral protein (S protein) plays a central role in the process of coronavirus jumping to humans [10].

SARS-CoV-2 was first discovered in December 2019. Lack of immunity to the new infection allowed the virus to reproduce freely and, as a result, mutate. In March 2020, Europe became epicenter of the novel coronavirus pandemic – a variant of the virus appeared that had an advantage over the wild-type virus: a single D614G mutation in the S protein made it possible to make the virus more transmissible. In April 2020, 1 million cases of COVID-19 were documented. The variant of the virus with D614G mutation continued to accumulate mutations rapidly changing the antigen (S protein) and increasing transmissibility of the virus. From December 2020, the new variants began to appear that were classified by the World Health Organization as variants of concern (VOC). Variant B.1.1.7 (Alpha variant) was first detected on December 14, 2020 in the UK. The next variant, B.1.351 (Beta variant), was discovered on December 18, 2020 in South Africa. The third variant, P.1 (B.1.1.28.1, Gamma variant), was identified on January 6, 2021 in Tokyo in a tourist from Brazil. In December 2020, variant B.1.617.2 (Delta variant) appeared, which replaced all other forms of the virus. Omicron variant (B.1.1.529) discovered in South Africa outcompeted Delta by November 2021 [11]. Omicron subvariants BA.1, BA.2, and BA.3 launched the fourth wave of COVID-19, followed by the fifth wave, which is still raging, formed by the lines BA.4 and BA.5, among which BA.5 still occupies a leading position. According to the European Center for Disease Prevention and Control (ECDC), by September 2022, the Omicron BA.2, BA.4, and BA.5 subvariants remained in the VOC category, with close attention being paid to the BA.4, BA.5, BA subvariants 2.75, and BA2.12.1. The reader can get information on phylogenetic tree of the origin of the main variants of SARS-CoV-2 at https://covariants.org.

## CORONAVIRUS ARCHITECTURE

**Fig. 1.**
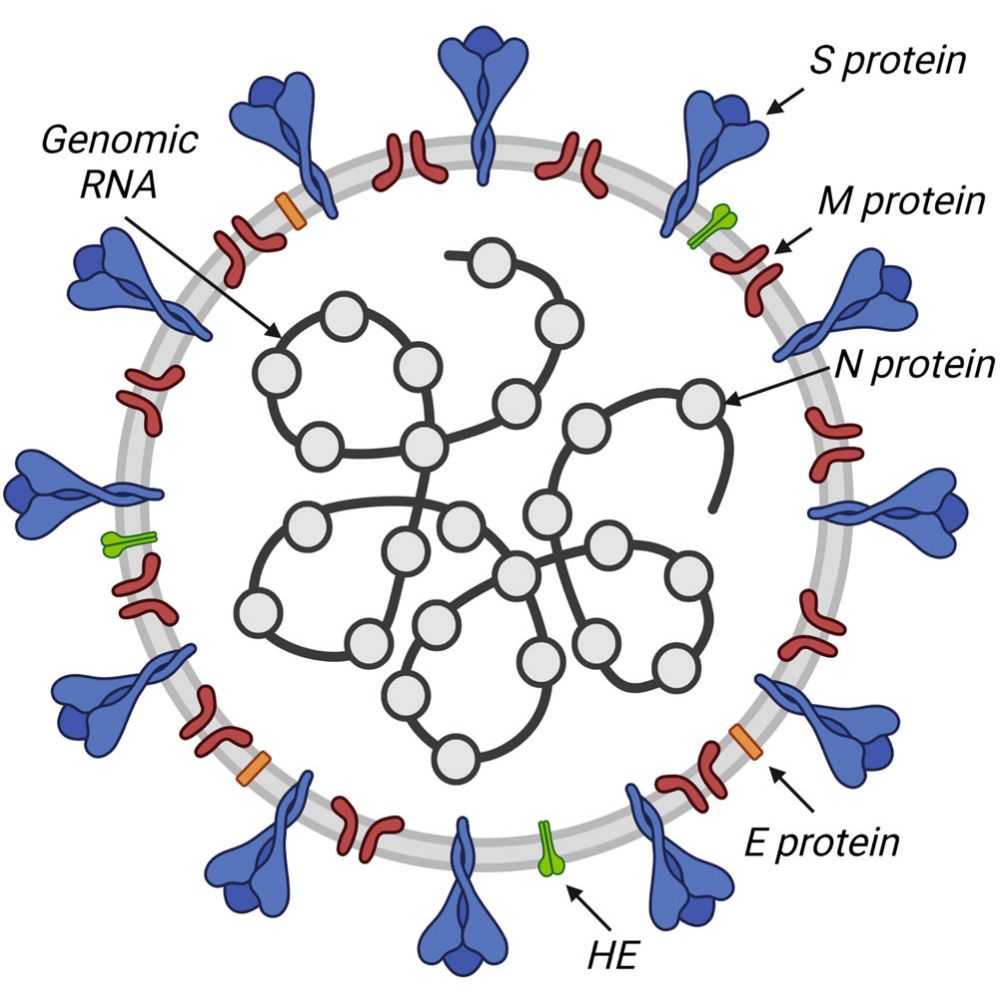
Coronavirus structure: N, S, M, E, HE structural proteins and genomic RNA are indicated.

Coronaviruses have a spherical shape of the virion with a diameter of 80 to 120 nm, framed by the so-called “spikes” – trimers of the Spike protein (S) (Fig. 1). In the HCoV-OC43 and HCoV-HKU1 coronaviruses, hemagglutinin esterase (HE) is also involved in formation of the so-called “crown”. The viral envelope is supported by the membrane protein (M) and contains small inclusions of the envelope protein (E). Under the virion envelope is a helically symmetrical nucleocapsid formed by a single-stranded genomic RNA coated with the nucleocapsid protein (N) [12].

S and HE are transmembrane proteins responsible for penetration of the virus into the cell. S protein (128-160 kDa) determines tropism of the virus; it binds to the receptors localized on the surface of the host cell. In HCoV-OC43 and HCoV-HKU1 viruses, the HE protein (48-67 kDa) facilitates attachment of the virus to the cell [13]. It has been shown for some viruses, that the presence of S protein on the surface of infected cell facilitates its fusion with the neighboring uninfected cell. Thanks to this strategy, giant multinucleated cells, or syncytium, are formed, function of which is to facilitate spread of the virus between the cells [14].

N protein determines architecture of the virus genome by forming a nucleocapsid with genomic RNA. Localized in the region of endoplasmic reticulum and Golgi apparatus, it is involved in the assembly and budding of the viral particles. N protein is also believed to be involved in regulation and modulation of replication and transcription. It has been shown that it could not only nonspecifically bind RNA, but also specifically interact with some sequences, including TRS (transcription-regulating sequences). Nucleocapsid proteins of various coronaviruses interact with a variety of other proteins, including nsp3 (non-structural protein 3) and host cell DDX1 RNA helicase. It has been suggested that the complex formed by DDX1 and phosphorylated nucleocapsid protein controls the balance between replication and transcription by modulating the level of template switching on TRS-B. The N protein of SARS-CoV-2 promotes association of RNA with the nsp7–nsp8–nsp12 complex and likely initiation of replication and transcription [15].

M protein is a core membrane protein. It is embedded in the lipid bilayer by three transmembrane domains; glycosylated ectodomain of the protein protrudes outwards. M protein maintains the viral envelope and determines the shape and size of the viral capsid interacting with other structural proteins. Interaction of M and S is necessary to retain S protein in the intermediate compartment between ER and Golgi apparatus (endoplasmic reticulum–Golgi intermediate compartment, ERGIC) and its inclusion in new virions. Binding of M and N stabilizes nucleocapsid as well as inner core of the virion and ultimately contributes to the completion of the virus assembly. Interaction of M and E is sufficient for the production and release of viral particles [2, 13].

E protein is a small integral protein (8-12 kDa), it is anchored into the membrane by the transmembrane domain, its ectodomain is glycosylated, and its endodomain is palmitized. For SARS-CoV and IBV viruses, E protein has been shown to form homopentamers that act as ion channels. Such structures modulate the process of virion release, taking an active part in the cell infection. Interestingly, E protein is produced in excess inside the infected cell, and only a part of it is included in the virion envelope, and most of it is located where the assembled viral particles accumulate and bud [14].

## STRUCTURE OF THE CORONAVIRUS GENOME AND MECHANISMS OF ITS EXPRESSION

**Fig. 2.**
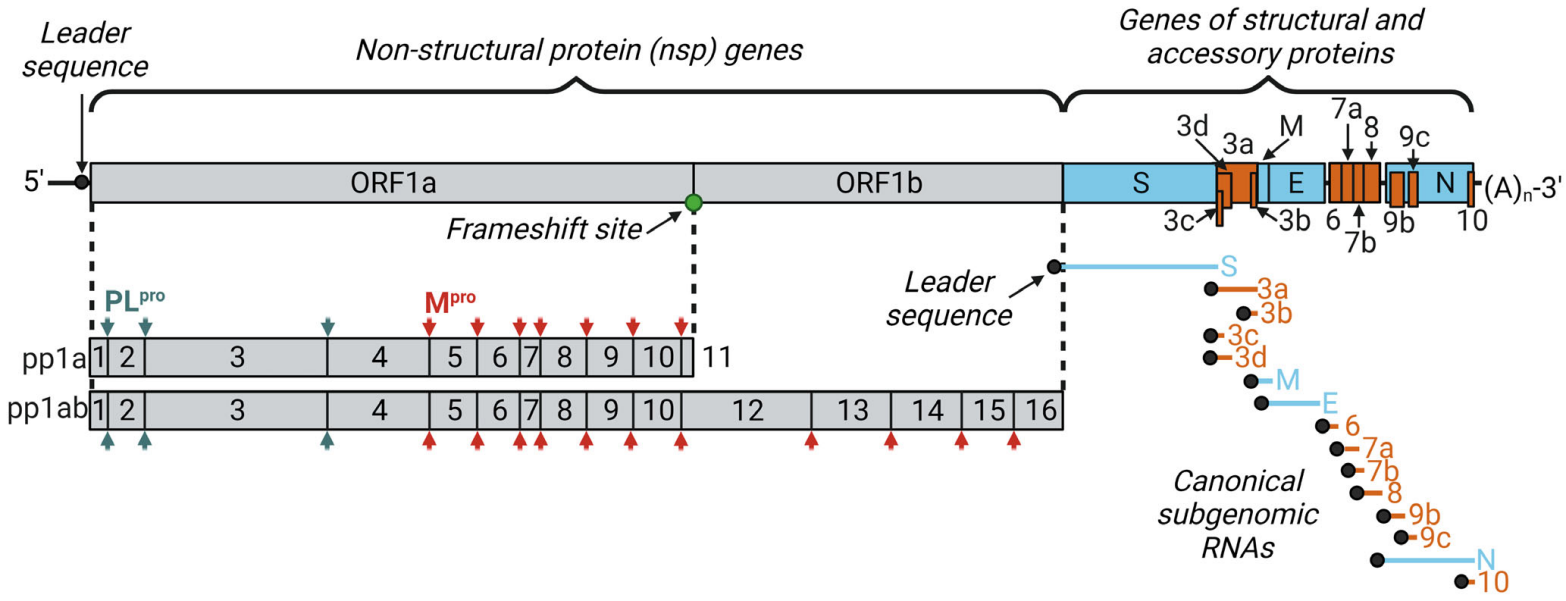
Schematic representation of coronavirus genome organization using SARS-CoV-2 as an example. Presence of the 5′-cap and a 3′-polyA tail at the ends of the genomic RNA allows immediate translation of nonstructural proteins from the ORF1a and ORF1b highlighted in gray. Reading frames are separated from each other by the reading frameshift site (slippery sequence). Translation results in two polyprotein chains, pp1a and pp1ab. Nonstructural proteins are formed as a result of proteolytic processing of pp1a and pp1ab by PLpro and 3-chymotrypsin-like proteases Mpro. Genes of structural and accessory proteins are transcribed into a set of subgenomic mRNAs. Genomic RNA and all subgenomic mRNAs contain the same leader sequence at their 5′-ends. Genes of structural and accessory proteins as well as their transcripts are highlighted in blue and orange, respectively.

Coronaviruses have the longest non-segmented genomes among all RNA virus [16]. The SARS CoV-2 genome is 26 to 32 kb, it encodes 16 non-structural (nsp1-16), 4 structural (S, M, N, and E), and 11 accessory proteins (ORF3a, ORF3b, ORF3c, ORF3d, ORF6, ORF7a, ORF7b, ORF8, ORF9b, ORF9c, and ORF10) [12, 17]. The carrier of genetic information of coronaviruses is the positive-sense single-stranded RNA. Each viral transcript, like genomic RNA, is capped at the 5′-end and polyadenylated at the 3′-end. This structure allows the cell ribosome to recognize genomic RNA as mRNA and immediately include it in the process of translation of viral proteins without adding transcription complex into the virion. In addition, all viral RNAs have a special leader sequence at their 5′-end, which distinguishes viral and cellular RNAs (Fig. 2) [18].

**Fig. 3.**
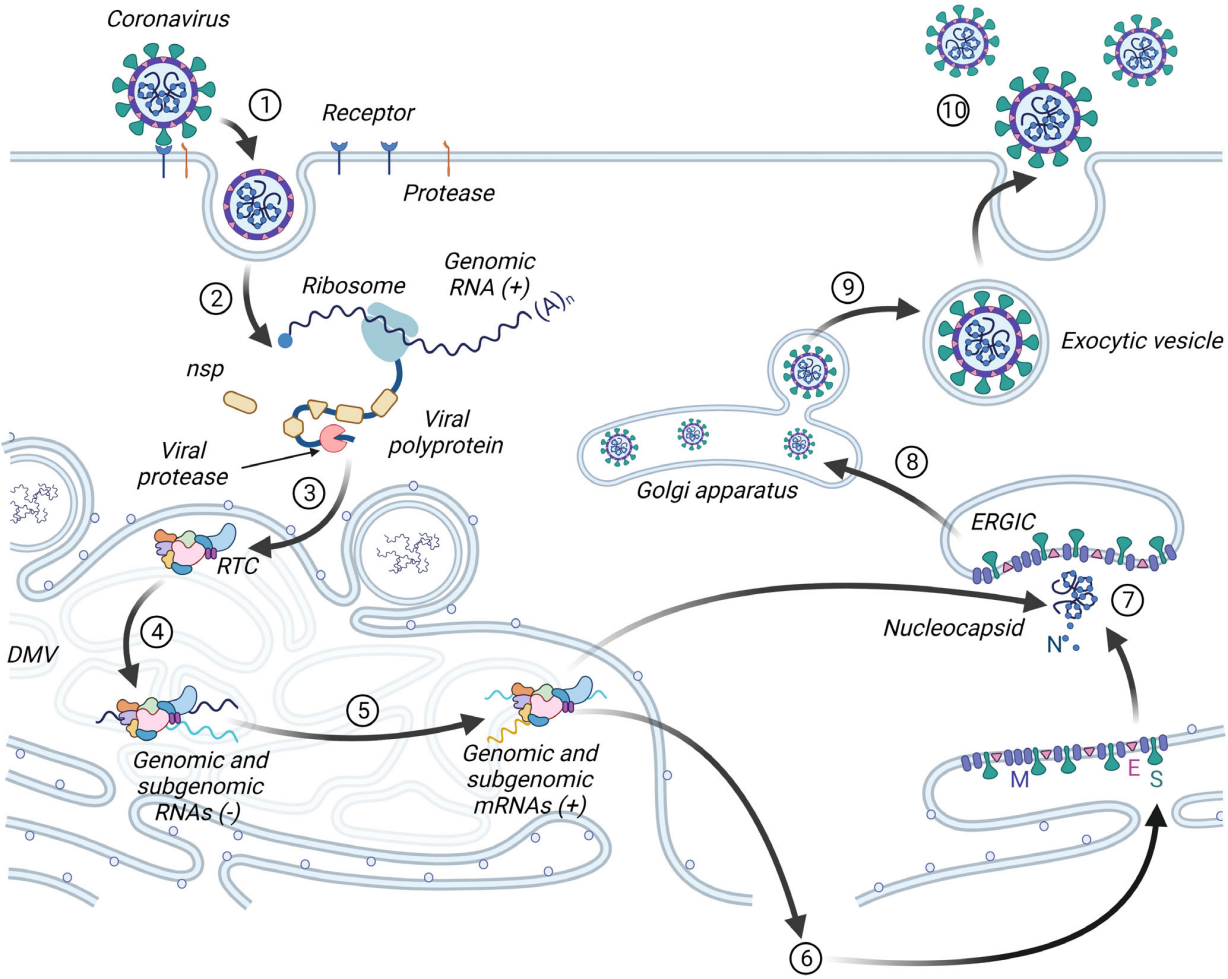
Life cycle of coronaviruses. Surface of the virus is coated with the S protein, which interacts with the receptor and activates fusion of the virus with the cell membrane after being cleaved by the cell surface protease (1). Genomic RNA, getting inside the cell, is immediately recognized by the ribosome, and translation of polyproteins and their processing to individual non-structural proteins occurs (2). Formation of the double-membrane vesicles (DMV) occurs in the membranes of endoplasmic reticulum with the replication–transcription complex (RTC) assembling in DMV (3). Genomic sense RNA is first converted into the antisense form to form genomic and subgenomic RNAs (4), and then into the sense form of the genomic RNA and subgenomic mRNAs (5). Subgenomic mRNAs are translated in endoplasmic reticulum into structural and accessory proteins (6). Genomic RNA interacts with N protein, forming a nucleocapsid (7), which combines with the structural proteins to form a virion (8). The mature virion (9) is released from the cell by exocytosis (10).

In the process of adaptation to eukaryotic cells, coronaviruses have developed different mechanisms for translating their genome. In particular, non-structural proteins are formed as two large polyproteins immediately after the virus enters the cell, which are further processed. For transcription of structural and accessory proteins, coronaviruses form special replication organelles – DMV (double-membrane vesicles), which are a reticulo-vesicular network of double-membrane vesicles with interconnected outer membranes (Fig. 3) [19]. Viruses produce DMV from the EPR membranes of the host cell by their successive rearrangements regulated by the non-structural proteins nsp3, nsp4, and nsp6, as well as proteins of the host cell [17]. The processes of replication and transcription of subgenomic RNA of coronaviruses take place in DMV replication–transcription complexes (RTCs) [20]. Formation of the replication organelles is characteristic of the RNA viruses with a sense chain of the genome in general. It is justified based on several facts: (i) there is accumulation of all factors of the virus and the host cell necessary for replication and transcription in one place; (ii) intermediate products of replication and transcription do not go beyond the DMV and are not recognized by the cellular antiviral response systems; (iii) there is a possibility of stricter coordination of the processes of replication and transcription within the DMV [21]. Along with the translation of nonstructural proteins in the cell, accumulation of the full-length antisense genomic copies occurs immediately, which are used as templates for formation of the new sense genomic RNAs. Synthesized sense genomes are either used to translate more non-structural proteins and RTC, or are packaged into the new virions. In addition, sense RNA is used to transcribe subgenomic RNAs by discontinuous transcription. First, the intermediate antisense subgenomic RNAs are formed, which are “translated” into the sense subgenomic mRNAs. Structural and accessory proteins are synthesized and moved first to the ERGIC – an intermediate compartment between the EPR and the Golgi apparatus, and then migrate to the Golgi apparatus, where formation of the mature virion occurs. The virion is released from the cell by constitutive exocytosis (Fig. 3) [22, 23].

## NON-STRUCTURAL PROTEINS: INTRACELLULAR SYNTHESIS AND FUNCTIONS

ORF1a and ORF1b reading frames make up two-thirds of the 5′-end. They encode information about all non-structural proteins of the virus (for SARS-CoV-2, this is nsp1-16), the genes of these proteins are fused to each other and do not have stop codons, with the exception of the site at the junction of the reading frames. Non-structural proteins determine replication of the virus and assembly of its transcription complex, in which transcription, processing, RNA modification, and correction of misplaced nucleotides take place.

**Fig. 4.**
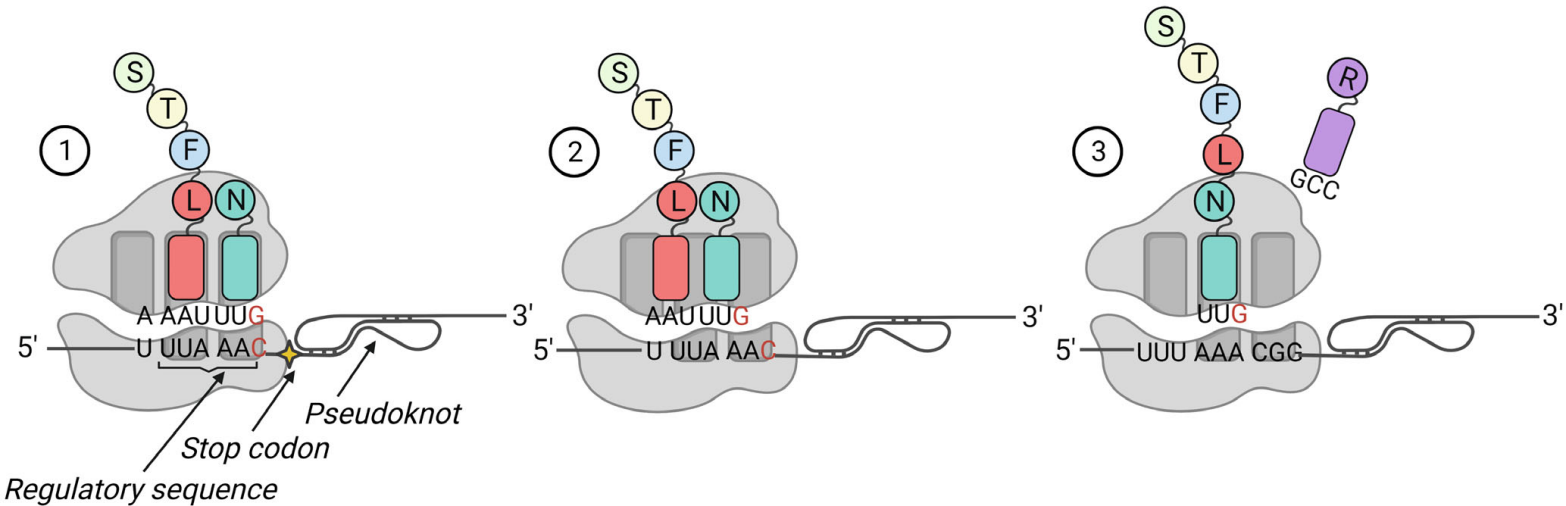
Frameshift during SARS-CoV-2 pp1ab synthesis. Not far from the regulatory sequence (slippery sequence) is a stop codon, followed by a stable RNA structure (pseudoknot) (1). When the ribosome approaches the pseudoknot, the rate of translation slows down, making it possible for the ribosome to jump back 1 nucleotide. This leads to the change in codon composition, which is associated with disappearance of the stop codon (2). The released third nucleotide of the triplet becomes the first in the new triplet. The corresponding tRNA approaches it, and protein translation continues (3).

Translation of the ORF1a and ORF1b results in generation of two polypeptide chains (pp1a and pp1ab). Stop codon located at the junction of the ORF1a and ORF1b reading frames allows the ribosome to complete its synthesis at this site with formation of pp1a (440-500 kDa). Sometimes the ribosome, having reached the special regulatory sequence (slippery sequence) X XXY YYZ (X – three identical nucleotides; Y – A/U; Z – A/C/G), located directly near the ORF1a stop codon, tends to slip out of the reading frame and jump to -1 nucleotide. In coronaviruses, this process is enhanced, since immediately after the regulatory sequence there is a stable RNA structure – a pseudoknot, bumping into which the ribosome pauses, and the probability of a jump increases (Fig. 4). If the frameshift occurs, then pp1ab (740-810 kDa) is formed, which is similar to pp1a in its N-terminus.

The internally located proteins are released after proteolytic cleavage by two cysteine proteases, papain-like proteinase (PL^pro^), and 3CL-like proteinase Mpro (major protease, 3CL^pro^), with the first located inside the nsp3 gene, and the second is nsp5. The papain-like protease processes nsp1, nsp2, nsp3, and also releases N-terminus of nsp4, while Mpro is responsible for processing of the most non-structural proteins: C-terminus of nsp4 and proteins nsp5-16 (Fig. 2) [23]. These proteases undergo autolytic processing before they begin to perform their functions [24, 25]. The pp1a polyprotein is cleaved into up to 11 proteins, while the pp1ab polyprotein is cleaved into up to 15 proteins [18]. Formation of several proteins from one reading frame makes it possible to accelerate production of viral proteins using the monocistronic principle of translation in a eukaryotic cell.

Arrangement of nonstructural proteins and their processing, apparently, reflects the need for these proteins in the process of genome translation. Once in the cell, the virus begins its “seizure” – synthesis of a large number of pp1a. When the cell functioning is modulated under the viral system and synthesis of the virus enzymes is necessary, there is a shift in the reading frame at the junction of ORF1a and ORF1ab leading to the synthesis of pp1ab. Next, assembly of the replicase-transcriptional complex, transcription of subgenomic RNAs, and their translation into structural and accessory proteins take place. It becomes possible to assemble virions that eventually leave the cell.

In the life cycle of coronaviruses, the non-structural protein nsp1 appears very quickly. It is responsible for suppressing expression of the host cell genes. By binding to the 40S subunit of the ribosome, it blocks translation of non-viral proteins, and, as a result, mRNAs of the host cell are degraded from the 5′-end. At the same time, translation of the viral RNAs continues because their 5′-ends are protected by leader sequences [18].

Nsp2-16 form the viral RTC and target specific subcellular regions, where they interact with the host cell factors that determine the course of replication cycle. Nsp2-11 aid accumulation of the viral RTCs by modeling intracellular membranes, they also are involved in immune system evasion and provide cofactors for replication. The process of RNA capping proceeds with participation of the nsp10 (it acts as a cofactor), nsp13 (has 5′-triphosphate activity), nsp14 (is an N7-methyltransferase), and nsp16 (has 2′-O-methyltransferase activity). The 3′-nontranslated region of coronaviruses contains the AAUAAA sequence, which serves as a polyadenylation signal for the nsp8 protein [26]. Nsp12-16 take on the main enzymatic functions: they are involved in the synthesis, modification, and correction of RNA (Table 1) [27]. Nsp15, a unique uridylate-specific endoribonuclease, shorten the poly-U regions that are present at the 5′-end of the viral antisense RNA, which aids innate immunity evasion [15].

**Table 1:** Functions of proteins involved in formation of the RNA-dependent RNA polymerase complex

Protein name	Participation in replication–transcription complex (RTC)
nsp7, nsp8	RNA polymerase holoenzyme subunits
nsp9	binding to RNA and to the replicase complex (presumably)
nsp10	activator subunit of the nsp14 exonuclease activity and nsp16 methyltransferase activity; regulation of ribosome frameshift
nsp12	RNA-dependent RNA polymerase, nucleotidyl transferase
nsp13	helicase, RNA 5′-phosphatase
nsp14	3′-5′ exoribonuclease, N^7^-methyltransferase
nsp16	RNA cap formation, ribose 2′-O-methyltransferase

The nsp12 protein represents the RNA-dependent RNA polymerase of coronaviruses. By itself, it has minimal polymerase activity, which increases significantly when interacting with the processivity factors nsp7 and nsp8. It is believed that the nsp12–nsp7–nsp8 axis is a RNA polymerase holoenzyme, the minimal complex required for polymerization of nucleotides [28]. Polymerase nsp12 is highly conserved; there is more than 95% homology among coronaviruses.

The nsp14 protein also interacts with the RNA polymerase holoenzyme of coronaviruses, due to which RNA is capped owing to methyltransferase activity of the enzyme. Its other very important function is the ability to correct erroneously inserted nucleotides owing to its 3′-5′-exonuclease activity, which is not characteristic of other RNA-containing viruses. The ability to correct erroneous nucleotides helps maintain integrity of the huge genome by reducing mutation rate of the error-prone RNA-dependent RNA polymerase.

**Fig. 5.**
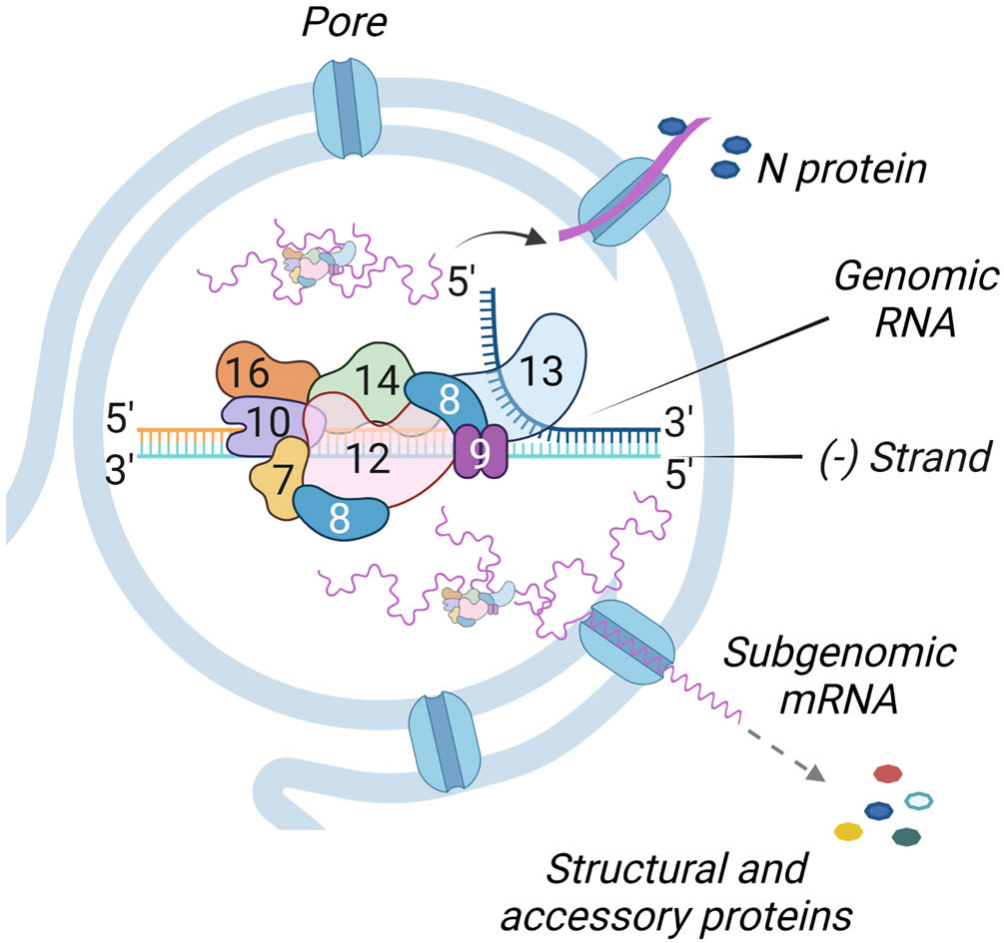
Replication and transcription of subgenomic RNAs take place within the double-membrane vesicle (DMV). The polymerase complex consists of nonstructural proteins nsp7, nsp8, nsp9, nsp10, nsp12, nsp13, nsp14, and nsp16 (indicated by numbers). RNA produced inside the DMV exits through the membrane pores. Subgenomic RNAs are translated into non-structural and accessory proteins; N protein meets with genomic RNA and forms a nucleocapsid.

Exonuclease activity is provided by the interaction of two proteins: nsp14 and nsp10, where nsp14 performs catalytic function and nsp10 activates it (Fig. 5). Sequence and structure of the exonuclease domain of nsp14 is very different from the known cellular exonucleases, which makes this enzyme an attractive target for the development of antiviral drugs [29].

## SYNTHESIS OF STRUCTURAL AND ADDITIONAL PROTEINS

Structural and accessory proteins are encoded in the last third of the coronavirus genome. For their translation, subgenomic messenger RNAs are formed in the infected cells. The virus receives these molecules in a special way – by “discontinuous” transcription, which occurs in coronaviruses and most members of the *Nidovirales* order, but has not been detected in other RNA viruses [30].

Viral sense RNAs at the 5′-end have the same leader sequence (TRS-L, TRS-leader), length of which varies in different coronaviruses from 55 to 92 nucleotides [31]. In the subgenomic mRNAs, this sequence is “fused” with another regulatory sequence, TRS-B (or TRS-body). In the genome of coronaviruses, the TRS-B sequence is located immediately before each open reading frame in the region of structural and accessory genes (with some exceptions), and TRS-L is located only in one place – at the 5′-end.

**Fig. 6.**
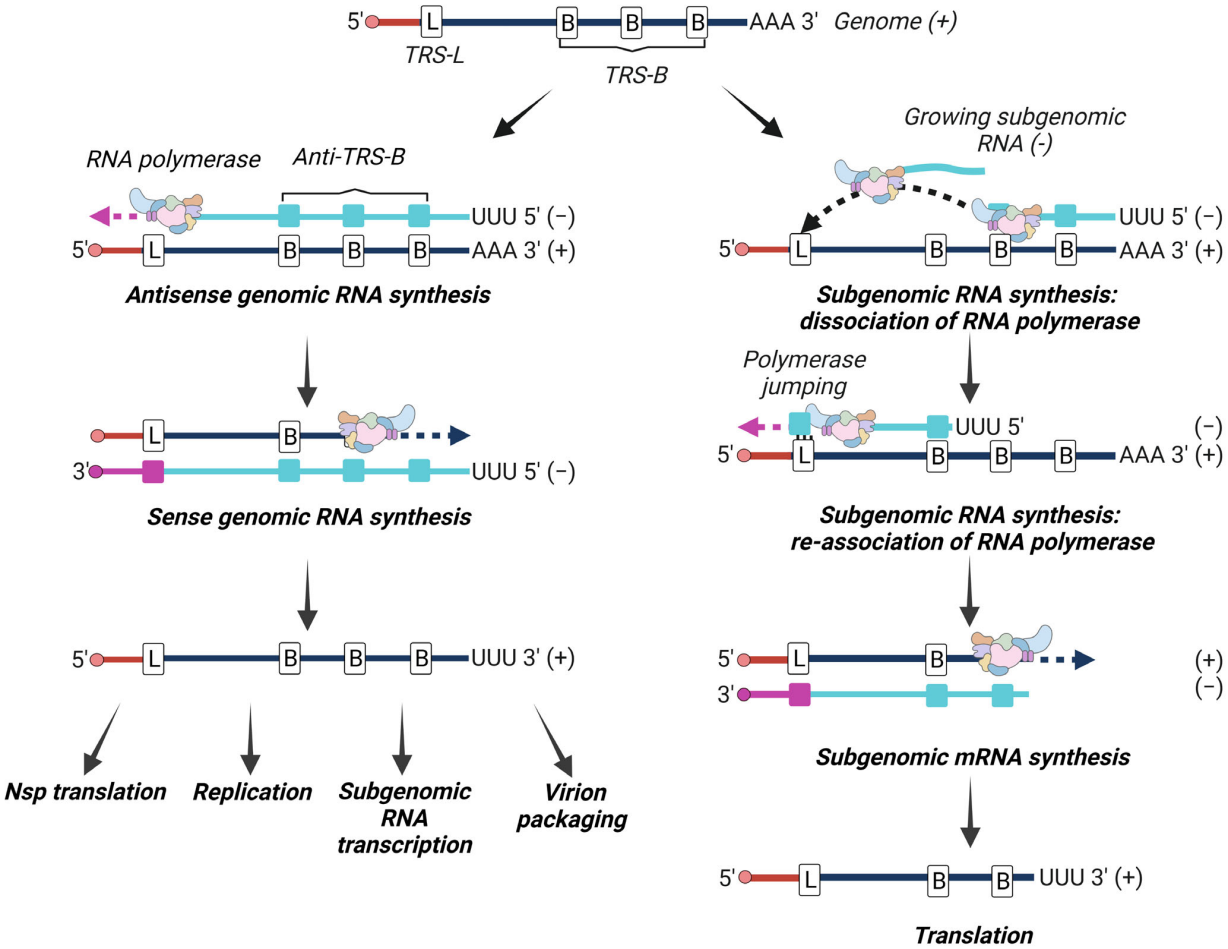
Replication and “discontinuous” transcription. Genomic RNA of the virus serves as a template for the synthesis of new copies of genomic RNA (left) and for transcription in subgenomic mRNAs according to the “discontinuous” principle (right). Copies of genomic RNAs are used for synthesis of non-structural proteins, replication, transcription into subgenomic RNAs, and packaging into a virion. Subgenomic mRNAs are translated into structural and accessory proteins.

During the synthesis of antisense strand, the RNA-dependent RNA polymerase reaches TRS-B, and after copying it stops its work and jumps to the beginning of genomic RNA in the TRS-L region to re-initiate synthesis. An interaction occurs between the complementary TRS: one is located on the nascent antisense RNA strand (TRS-B), and the second is located on the messenger sense genomic RNA (TRS-L). TRS interaction occurs through the conserved sequence (5′-ACGAAC-3′ for SARS-CoV and SARS-CoV-2) that is surrounded by variable length and variable sequences that could also facilitate TRS-B and TRS-L interaction [15]. After reinitiation of the RNA synthesis on TRS-L, a copy of the leader sequence located on the template strand is added to the nascent RNA, and synthesis of the antisense subgenomic RNAs is completed. These intermediate RNAs are then used to transcribe subgenomic mRNAs that have a sense strand and are used as mRNAs to translate structural and accessory proteins (Fig. 6) [18]. The set of subgenomic mRNAs have the same 3′- and 5′-ends, but vary in their internal regions. Such RNAs are considered polycistronic, but they are functionally monocistronic because translation is limited to the single open reading frame closest to the 5′ leader sequence [15].

The number of separate subgenomic RNAs synthesized in the infected cells depends on the location and reading efficiency of each TRS-B [26]. It has been shown that the ribosome seating density determines the number of transcripts from a given TRS [15].

For the most representatives of coronaviruses, from 5 to 8 subgenomic RNAs have been identified, 4 of which encode the obligatory structural proteins of the virion (S, E, M, and N), and the rest – accessory proteins. In the cells infected with SARS-CoV-2 and SARS-CoV, 11 and 10 subgenomic mRNAs have been identified, respectively [17].

In addition to the canonical subgenomic RNAs, other non-canonical RNA products of discontinuous transcription were found [31]. And there is a lot of intrigue here: there are those in which 5′-leader sequences are attached to unexpected 3′-sites, TRS-L-independent long hybrids, and products resulting from deletions of structural and accessory genes [15]. Non-canonical subgenomic RNAs can account for up to 1/3 of the total number of subgenomic RNAs [32].

The authors of [33] studied the SARS-CoV-2 variant containing the D614G mutation and three adjacent nucleotide substitutions spanning over two residues of the nucleocapsid protein (R203K/G204R; variant B.1.1). Sequence analysis suggests that these substitutions are the result of homologous recombination of the TRS-L core sequence. Due to this, a new TRS appeared between the RNA binding domains and the nucleocapsid dimerization domains, which led to the emergence of a new subgenomic RNA transcript. Viruses with the K203/R204 mutation may also overexpress subgenomic RNA from other open reading frames. The ability of SARS-CoV-2 to introduce new TRS motifs into its genome, with the potential for new subgenomic RNA transcripts, suggests that this is a way to increase efficiency and adaptation. This study also suggests that SARS-CoV-2 itself may act as a source of homologous recombination [33].

Many non-canonical subgenomic RNAs have coding potential and their products may be truncated versions of the accessory proteins or may themselves be unexplored proteins. Using the example of SARS-CoV-2, a subgenomic RNA has been identified that could be translated into the S protein truncated from the N-terminus (deletion of 143 amino acids). A similar protein has been identified in the porcine respiratory coronavirus [31]. Davidson et al. [34] presented the evidence based on the peptide mapping obtained with the help of tandem mass spectrometry, indicating presence of the previously unknown viral proteins, some of which may originate from the non-canonical subgenomic RNAs. [34]. Also, non-canonical subgenomic RNAs could function as defective interfering RNAs [35].

Presence of non-canonical RNAs has been shown for various coronaviruses (e.g., MHV, HCoV-229E, SARS-CoV-2). It is not clear yet whether these non-canonical RNAs are synthesized as a result of unusual discontinuous transcription (although the use of TRS-like sequences for the synthesis of subgenomic transcripts has been shown [15]), or they are the products of recombination.

TRS are thought to represent recombination “hot spots” and secondary RNA structures promote TRS template switching in a TRS-independent manner. Transcriptomics and ribosome profiling experiments have demonstrated a complex landscape of SARS-CoV-2 RNA and potential proteins that goes far beyond the “canonical” gene expression program of coronaviruses. Similar observations have been made for other coronaviruses [15]. A detailed study of these processes is yet to come.

At the moment, it has not been clearly established whether the compositions of the RTCs that perform synthesis of antisense genomic RNAs and subgenomic RNAs are identical. Probably, the balance between replication and transcription is determined by interaction with the specific protein factors.

Accessory proteins are the most unexplored elements of coronaviruses. They are not important participants in the replication process, however, apparently, they play an important role in pathogenesis of the virus, that is, in the interaction of the virus with the host organism. Most of the functions attributed to the accessory proteins are related to the mechanisms of evasion from the action of immune system of the host organism. Such functions include, for example, inhibition of cytokine secretion with participation of ORF9c or antagonism of the type 1 interferon with participation of ORF3b, ORF6, ORF7a, ORF8, or ORF9b. In addition, these accessory proteins (e.g., ORF3a) influence important cellular processes such as autophagy or apoptosis. ORF3b affects functioning of mitochondria, and ORF9b activates inflammation [17]. Many functions of these proteins remain unknown due to insufficient homology with the known proteins [23].

## MECHANISM OF THE VIRUS ENTRY INTO THE CELL

Coronaviruses enter the cell following two successive events: (i) viral S protein interacts with its receptor on the cell surface, and next (ii) S protein is transformed into its active form capable of stimulating fusion of the viral and cell membranes by cleavage with the intracellular protease.

Four receptors have been described that are used by coronaviruses to bind to a host cell. S proteins of the HCoV-229E coronavirus, porcine transmissible gastroenteritis virus (TGEV), and feline infectious peritonitis virus (FIPV) interact with aminopeptidase N (APN, CD13). Moreover, in order to transform the pig aminopeptidase into receptor of the human HCoV-229E virus, it is sufficient to change only 8 amino acids in the hypervariable region of the receptor-binding domain (RBD) [36]. The CEACAM1 adhesion molecule is the entry receptor for the murine hepatitis virus (MHV), which belongs to the genus of Betacoronavirus. The RBD of S protein of this virus is located in an unusual location for coronaviruses, in its N-terminal domain, where it functions as a lectin, binding carbohydrate residues. This allowed the ancient coronavirus to expand its tropism and increase infectivity. SARS-CoV, SARS-CoV-2, some SARS-related bat viruses use the angiotensin-converting enzyme ACE2 to enter the cell. MERS-CoV, camel MERS-CoV, and BatCoV-HKU4 use DPP4 protein receptor [10].

Three of these receptors (APN, ACE2 and DPP4) have peptidase activity. However, it has been shown that proteolytic activity of these molecules is not essential for the successful binding and entry of coronaviruses. Binding of the RBD and the receptor occurs on the outer side of the receptor, without affecting its catalytic site. However, presence of the transmembrane proteases (for example, TMPRSS2) is critical [10].

S protein (180-200 kDa, 1273 amino acids) is located on the surface of the virion in the form of a trimer, forming a convex “head” and a “stem”. Each trimer is coated with 66 glycans, which prevent recognition of the virus by the host’s immune system and help the virus to attach. There are 2 subunits in the S protein: S1 is responsible for binding to the receptor, and S2 is responsible for fusion of the virus with the host cell membrane. Main domains of the S1 subunit are the N-terminal domain (NTD) and RBD. Within the RBD domain, the RBM motif is recognized, which directly binds to the receptor. RBD-domains of the S protein trimer can exist in two different conformations: open (up-conformation, the state accessible to the receptor) and closed (down-conformation, the state of inaccessible to interaction with the receptor) [37]. Fusion peptide (FP), two domains (HR1 and HR2, heptad repeat), transmembrane (TMD), and cytoplasmic (CTD) domains are identified in the S2 subunit. Using ACE2 as an example, it has been shown that 2 trimers of S protein bind to one receptor dimer, which accelerates the process of virus penetration.

In the native conformation, the S1 subunit “wraps” the S2 subunit, which forms center of the protein. After the S1 subunit is cleaved, large-scale rearrangements occur within the S2 subunit, including refolding of the HR1 domain, due to which the FP fusion peptide is released and integrated into the host cell membrane [37].

S protein contains two regions, sequential cleavage of which leads to its activation on the cell surface. The first site, S1/S2, is located at the junction of two subunits. In some, but not all, coronaviruses, it is recognized by proteases located on the cell surface and cathepsins. MERS-CoV, IBV, SARS-CoV, SARS-CoV-2 are examples of viruses that cleave at the S1/S2 site [38]. Compared to other coronaviruses, SARS-CoV-2 has an evolutionary advantage – insertion of the polybasic cleavage site in this region, which, in turn, is a minimum recognition site for furins, peptidases abundant in epithelial cells. This allowed SARS-CoV-2 to use a wide range of different proteases to activate the cell entry process. The second site is located within the S2 subunit and is called S2′. Its cleavage can take place in the extracellular space or on the cell surface with participation of serine proteases such as trypsin, TMPRSS2, or neutrophil elastase. In the absence of S1/S2 cleavage, S2′ activation is still possible by cathepsins (cathepsin L) at the time of virion maturation during the passage of the endocytic pathway. That is, coronaviruses can fuse with the cell plasma membrane or endosomes using an early and a late pathway. The early pathway involves cleavage first at the S1/S2 site and next at the S2′ site by extracellular and cellular transmembrane proteases (trypsin, neutrophil elastase, TMPRSS2), while in the late pathway the process is driven by endosomal proteases such as cathepsin L [39].

**Fig. 7.**
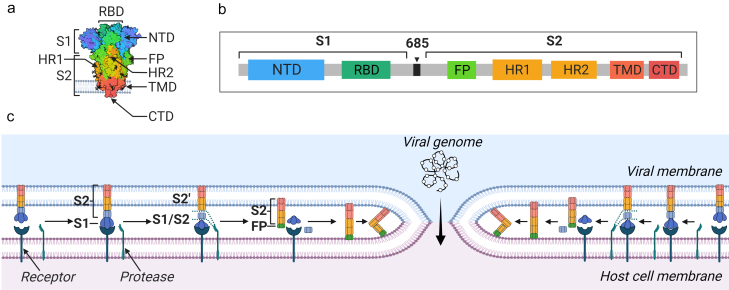
Domain organization of S protein and its participation in the fusion of a viral particle with a cell. 3D (a) and primary (b) structure of the SARS-CoV-2 S protein. The following domains are recognized within the S1 and S2 subunits: NTD, N-terminal domain; RBD, receptor-binding domain; FP fusion peptide; HR1 and HR2 domains; transmembrane and cytoplasmic domains. c) Scheme of interaction between a cell and a coronavirus particle. S protein located on the surface of the viral membrane finds the corresponding receptor on the cell membrane. After their interaction with each other, cellular protease cleaves the S protein at the S1/S2 and S2′ sites. This results in the release of the activated S2 subunit with the FP fusion peptide protruding towards the cell. The peptide is integrated into the cell membrane, the HR1 and HR2 domains interact with each other, “pulling” both membranes close to each other.

After cleavage at the S2′ site, the FP fusion peptide is exposed to the outside and triggers fusion with the host cell membrane. Hydrophobic interactions then occur between the HR1 and HR2 domains of the S2 subunit, resulting in formation of a six-chain structure that causes the membranes of the virus and the cell to approach each other forming a pore. The pore size increases until genetic material of the virus enters the cell (Fig. 7).

## SARS-COV-2 MUTATIONS

Evolution of the virus is ongoing. It is believed that mutations occur and become fixed especially intensively during the long-term persistence of the virus in the human body with weakened immune system [40]. Mutations and their combination modulate virulence or contagiousness, affect clinical picture and severity of the consequences of COVID-19 (Table 2).

**Table 2:**
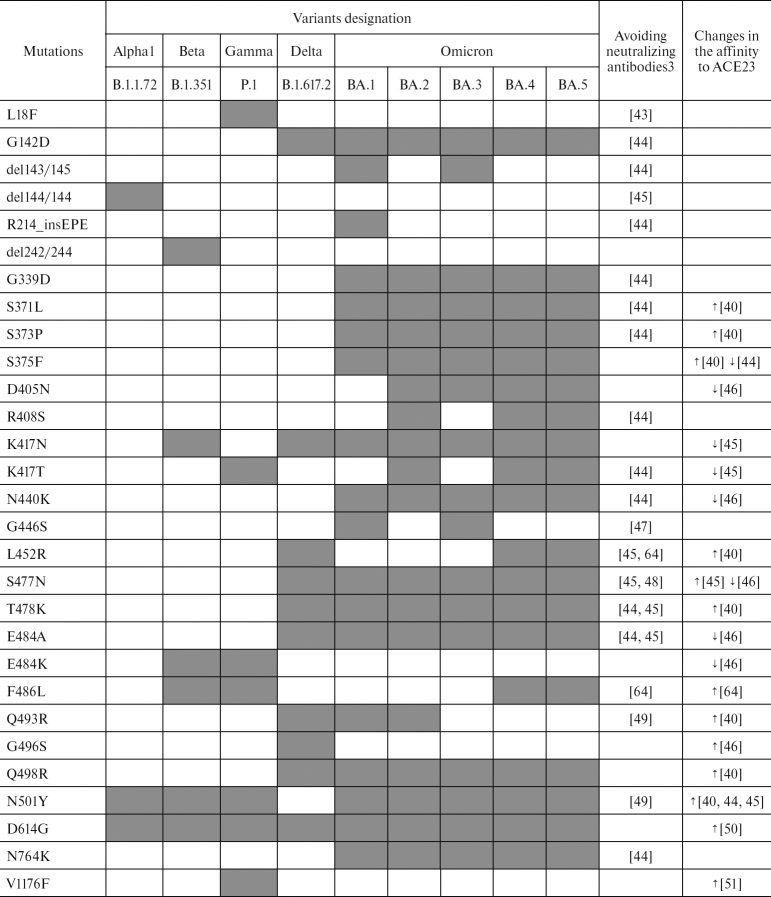
Mutations in the S protein of SARS-CoV-2 VOCs [41, 42] affecting immune response and affinity to ACE2 receptor Note. Gray background indicates presence of mutation that changes response to neutralizing antibodies or binding affinity to ACE2; colorless background indicates absence of mutations. ^1^ WHO designation. ^2^ Pango designation. ^3^ The column contains references to literary sources, which describe effect of the mutations on the ability of the virus to avoid neutralizing antibodies or changing its affinity to ACE2 (increase ↑ or decrease ↓).

At the beginning of formation of SARS-CoV-2 variants, the D614G mutation in the S protein gene [52] contributed to the rapid spread of the virus and became the first mutation that was preserved in all variants that appeared after first emergence in Wuhan [53]. D614G is characterized by the replacement of aspartic acid with glycine at position 614 in the S protein. Residue 614 is located at the interface between the S1 and S2 subunits and may affect their interaction and stability of the S protein. A loop disordered in the D614 S-trimer wedged between the domains within the protomer in the G614 spike. This additional interaction seems to prevent premature dissociation of the G614 trimer, which results in the S protein becoming more stable than in the original version of the virus. Presence of this mutation is associated with high viral load in the infected patients and high infectivity in the *in vitro* model of viruses pseudotyped according to the VSV∆G*/GFP system. At the same time, presence of the D614G mutation does not correlate with severity of the disease [54].

For the Delta variant, four critical mutations, which affect characteristics of the virus have been described: D614G and G142D increase affinity for ACE2, as well as L452R and T478K provide high affinity and inability of antibodies to neutralize the virus (Table 2). These mutations are not unique and occur in other variants, but their combination in the Delta variant could determine long and severe course of the disease. In the paper published in the journal *Science*, two characteristics of the Delta variant were described, explaining its higher transmissibility [55]. Firstly, even at low levels of ACE2, the Delta variant S protein fuses efficiently with the cell. Secondly, infection of the target cells with this variant occurs much faster than with the viruses of another variant. Thus, with a relatively short exposure, Delta variant can quickly infect many more cells, resulting in a short incubation period and a higher viral load during infection [55].

Planas et al. studied sensitivity of the Delta variant to monoclonal antibodies and antibodies present in the blood serum of individuals who recovered from COVID-19 or were vaccinated against COVID-19. Several anti-NTD and anti-RBD monoclonal antibodies, including bamlanivimab, have been shown to have lost their ability to bind to the S protein and were no longer able to neutralize the Delta variant. Sera collected from the patients recovered from COVID-19 12 months after onset of the symptom were four times less effective against the Delta variant than against the Alpha variant (B.1.1.7). Sera from the individuals who received a single dose of Pfizer or AstraZeneca vaccine showed only a slight inhibitory effect on the Delta variant. Sera from the individuals who received both doses of the vaccine exhibited a neutralizing effect against the Delta variant, however were about three to five times less effective than against the Alpha variant [56]. In another study, two doses of the vaccine were shown to provide the best protection, but this also depended on the variant of the virus. Thus, effectiveness of the Pfizer vaccine against the Delta variant is reduced to 88%, while for the Alpha variant its effectiveness is 93.4%. Vaccination with AstraZeneca was effective in 66.1% of the cases with the Alpha variant and 59.8% with the Delta variant [57].

The ubiquitous and rapid spread of the Omicron variant has led to the significant virus evolution within this VOC. A particularly large amount of information on the mutations in this variant has been collected regarding the ability of avoiding neutralizing antibodies and change of the affinity for the receptor (Table 2). In this variant we are able to observe a significant number of mutations located in different parts of the genome. In the S protein of Omicron, the number of mutations is more than 30 [58] with more than 10 located in the RBD domain. According to some reports, existence of a large number of mutations in the virus of this variant indicates its origin from immunocompromised people [40].

The affinity-altering mutations within the same Omicron variant/sub-variant often have opposite effects and can neutralize each other, which may be the reason for the rather mild COVID-19 caused by this variant. At the same time, the ability to avoid neutralizing antibodies could be the reason for easier spread of this variant between people: the Omicron variant B.1.1.529 is 3.3-fold more transmissive than the Delta variant [59]. Many Omicron variant mutations occur in the previously discovered strains of SARS-CoV-2. The unique mutation of the BA.1 subline is the insertion R214_insEPE. This insertion appears to have resulted from recombination occurring in the people infected with multiple variants of coronaviruses at the same time. An identical sequence has been established in the S protein of HCoV-229E that can be used for matrix switching [60].

Mutations found in all sub-variants of Omicron are G142D (except BA.1, which has G142), G339D, S371L, S373P, S375F, K417N, N440K, S477N, T478K, E484A, Q498R, N501Y, Y505H, D614G, H655Y, N679K, P681H, D7964K, Q954H, and N969K). Among these, the N501Y and Q498R mutations are thought to enhance binding to the ACE2 receptor, and the H655Y, N679K, and P681H mutations increase S protein cleavage and facilitate virus transmission [42]. The T9I mutation of the E protein, observed in all sub-variants of Omicron and located in the transmembrane domain, could affect configuration of this protein, providing stronger anchoring of the viral membrane [61]. Several critical RBD binding sites for the ACE2 receptor were identified in the Beta and Omicron variants: K417N, E484K, Q493H, N501Y for the Beta strain and Q493R, Q498R, N501Y for the Omicron strain. It is hypothesized that mutations at these sites may lead to the expansion of the tropism of this virus [62, 63].

S protein of the BA.4 and BA.5 subvariants dominant as of September 2022 is identical and very similar to the BA.2 spike protein. The difference is that these two sub-variants have a 69-70 deletion, in addition, the L452R mutation characteristic of the Delta variant, the new F486V mutation, and the R493Q reverse mutation. Other parts of the genome have additional mutations. For example, subvariant BA.4 is characterized by the mutations L11F in ORF7b, P151S in the N protein gene, deletion 141-143 in nsp1, while BA.5 has the D3N mutation in the M protein gene [64].

Substitutions in the S protein at positions 452, 486, and 493 are thought to alter ACE2 binding and affect antibody interactions. Mutations at position L452 with substitutions for R/M/Q occurred in Delta, Kappa, and Epsilon variants, but also independently occurred in several BA.2 sub-variants at different geographic locations (eg, L452Q in BA.2.12.1). Mutation at this position impairs the ability of antibodies to neutralize the virus [64]. The amino acid at position F486 is involved in the binding of the virus to the ACE2 receptor. Mutation at this position results in the decrease of neutralizing activity of the class I and II antibodies and polyclonal serum. It is believed that F486 gives the virus a great advantage in avoiding the action of neutralizing antibodies, including antibodies that neutralize BA.1. Hence, the BA.4 and BA.5 sub-variants acquired even more effective antibody evasion mechanism than the previous sub-variants [64]. In addition, BA.5 exhibits the highest transmissibility as of September 2022 according to the weekly WHO updates. BA.2.12.1 and BA.2.75 are also derived from BA.2. The L452Q and S704F substitutions are present in the region of the BA.2.12.1 S protein, and the K147E, W152R, F157L, I210V, G257S, D338H, G446S, N460K substitutions and the reverse mutation Q493R are present in BA.2.75.

A few months after the start of COVID-19 pandemic, recobinant genomes began to be detected in the samples form infected individuals. In general, this was predictable – homologous and non-homologous recombination occurs quite often in coronaviruses. Homologous recombination is believed to proceed according to the general scheme with cleavage of the RNA-dependent RNA polymerase from the template during RNA synthesis and its attachment to the homologous site of the template of another genome, followed by elongation. Non-homologous recombination can occur between the genomic RNA and subgenomic mRNAs as a result of collapse of the transcription complex during discontinuous transcription. In order for recombination event to take place, simultaneous circulation of two or more subvariants in the population and appearance of different genomes inside one cell are necessary.

For the first time, a genome derived from two different variants of SARS CoV-2 (20A and 20B according to the NextStrain classification) was reported in February 2021 [65]. Later, various hybrid genomes were often detected among the sequenced sequences. The detailed description of more than twenty variants of fixed hybrid genomes obtained as a result of recombination events (hybrids Alpha-Delta, Beta-Delta, Delta-BA.1, BA.1-BA.2) can be found in [66]. The recombinant XD variant is the Delta genome, which acquired the BA.1 S protein sequence (nucleotides at positions 21643 to 25581). XD contains a unique NSP2 mutation: E172D [67]; Line XF, which is a recombinant of Delta variant AY.4 and BA.1 with a break point near the end of nsp3 (nucleotide 5386), where fragments of two different genomes are located sequentially one after another; the XE recombinant combines the genomes of BA.1 and BA.2, while most of the genome, including the S protein gene, belongs to BA.2. Hybrids of the latest variants of SARS-CoV-2 have also appeared, for example, BA.2.12.1-BA.5; BA.2.76-BA.5.2; BA.5-BA.2-BA.5.1 [66].

These variants are not currently considered to pose a threat of higher transmissibility or worsening of the course of the disease (Global Virus Network, https://gvn.org/covid-19), due to the facts that the pandemic waves of the latest variants of the virus become shorter minimizing the time of simultaneous circulation of different lines; high reproduction index of the latest variants of the virus sets a high bar for recombinant forms; due to the mild course of the disease caused by Omicron, the time for virus replication within the body is limited, making the likelihood of co-infection or superinfection minimal.

## CONCLUSION

The mechanisms of gene replication and transcription, interaction of coronavirus proteins with human receptors have previously been studied using SARS-CoV and MERS-CoV as examples. But lack of the drugs for effective treatment of coronavirus infections allows us to conclude that not all molecular mechanisms of the functioning of coronaviruses have been identified. The study of SARS-CoV-2 has improved our understanding of the mechanisms of virus entry into the cell, helped to determine structure and functions of the coronavirus proteins, to identify key mutations that affect contagiousness and transmissibility of the virus, as well as clinical manifestations of the disease. For SARS-CoV-2, its unique ability to shift the reading frame during transcription of its genomic RNA described in detail results in the increased variability of the synthesized proteins. These proteins are able to “build” intracellular vesicles, where replication and transcription of the viral RNA takes place, as well as subsequent assembly of the viral particles. Syncytium formation has previously been shown for some viruses, such as HIV and herpes simplex virus. However, the mechanisms by which such structure is formed were largely unexplored until the advent of SARS-CoV-2. For example, it has been shown that the ability of cells to merge during the syncytium formation is determined by the presence of a unique to the SARS-CoV-2 S protein at the junction of S1 and S2 subunits.

Critical and multi-role involvement of the S protein in the SARS-CoV-2 pathogenesis is very interesting in itself. It is involved in fusion with the cell, formation of syncytium, avoiding immune response; glycans on its surface protect the virus from rapid recognition by the immune system. Nucleotide sequences of the S protein of the SARS-CoV, MERS-CoV, and SARS-CoV-2 viruses are very similar. However, response of the human body to each of these viruses is different. The COVID-19 pandemic has given us the opportunity to witness evolution of the virus first hand. We have witnessed how the outcome of the disease differs greatly from the number and combination of mutations in viral variants. Amino acid composition of the S protein determines efficiency of the virus penetration into the cell, its pathogenicity, transmissibility, and evolution.

At the moment, it is impossible to determine true scale of the global consequences caused by the SARS-CoV-2 virus pandemic, both for the healthcare system and for the social and economic spheres as a whole. We can definitely say that in the next decade we will observe and study the consequences of coronavirus infection, evaluate changes in medical statistics, study the risks of delayed complications, such as the expected increase in autoimmune and neurodegenerative diseases; analyze trends and risks for patients with chronic and oncological diseases, study health status of pregnant women and children infected with SARS-CoV-2 during the gestational period. A special group of studies will include work on the assessment of neurological consequences associated with social distancing, including in children and adolescents. In addition, likelihood of the evolution of the SARS-CoV-2 virus remains a hot issue. Theoretical possibility of the virus evolution and combination of the transmissibility of SARS-CoV-2 with the lethality of MERS-CoV raises the issue of globalization and development of new methods to combat viral infections before the world scientific community. The need to track and control forms of coronaviruses in nature has become obvious as well as the need for reform in registration, approval, and implementation of medical devices for treatment of infectious diseases, but most importantly, there is a need for creation of a dynamic environment with free access to the latest scientific tools and highly specialized scientific and medical personnel to quickly respond to biological challenges in future.

## Abbreviations

ACE2: angiotensin-converting enzyme 2
DMV: double-membrane vesicle
E: E protein, SARS-CoV-2 envelope protein
HCoV: Human coronavirus
HE: hemagglutinin esterase
HR: heptad repeat
M: M protein, SARS-CoV-2 membrane protein
MERS: Middle East respiratory syndrome
N: N protein, SARS-CoV-2 nucleocapsid protein
nsp: non-structural proteins
ORF: open reading frame
pp: polyprotein
RBD: receptor-binding domain
SARS: severe acute respiratory syndrome
S: S protein, SARS-CoV-2 spike protein
TRS: transcription-regulating sequence
S1 and S2: SARS-CoV-2 spike protein subunits
